# Activation of HIV-1 expression and replication by cGMP dependent protein kinase type 1-β (PKG1β)

**DOI:** 10.1186/1742-4690-4-91

**Published:** 2007-12-13

**Authors:** Jia Hai Lee, Venkat RK Yedavalli, Kuan-Teh Jeang

**Affiliations:** 1 Molecular Virology Section, Laboratory of Molecular Microbiology, National Institute of Allergy and Infectious Diseases, National Institutes of Health, Bethesda, Maryland 20892-0460, USA

## Abstract

The effect of cGMP (cyclic GMP) dependent protein kinase 1-β (PKG1-β) and cGMP analogues on transcriptional activity and replication of human immunodeficiency virus type 1 (HIV-1) was investigated. Transfection of PKG1β expression plasmid increased expression from an HIV-1 LTR-reporter as well as from an infectious HIV-1 molecular clone, pNL4-3. Treatment of HIV-1 AD8-infected monocyte derived macrophages (MDMs) with cGMP agonists and cGMP antagonists caused respectively increased and decreased virus replication. These findings provide evidence that cGMP and PKG serve to regulate HIV-1 infection in human cells.

## Findings

Previously nitric oxide (NO) was postulated to have a negative effect on HIV-1 replication through a cGMP-independent route [1]. However, it was not characterized as to how this cGMP-independent effect manifested mechanistically. On the other hand, it is well-accepted that a major intracellular signaling pathway for NO is through a cytosolic-guanylate cyclase linked cGMP-dependent protein kinase, PKG, pathway [2]. cGMP/PKG has been shown to activate abundantly both CREB [3] and NF-κB [4,5]. Interestingly, to our knowledge, no systematic investigation of cGMP/PKG's activity on the HIV-1 LTR has been reported to date.

**Figure 1:**
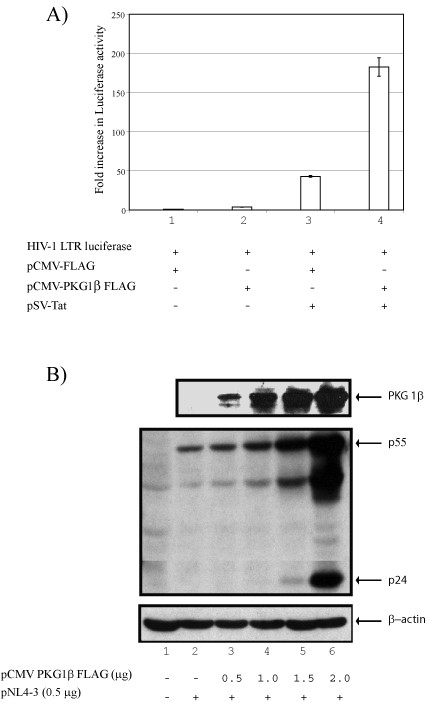
PKG1β activates expression from the HIV-1 LTR. A) Activation of HIV-1 LTR luciferase by PKG1β. HeLa cells were cultured in medium with 0.1% fetal bovine serum and transfected as indicated with HIV-1 LTR luciferase reporter plasmid in the absence and presence of Tat, and with a PKG1-β expression plasmids. PKG1β was found to increase Tat dependent transcriptional activity of the HIV-1 LTR by four fold. B) Co-transfection of PKG1β and HIV-1 infectious molecular clone, pNL4-3, resulted in dose dependent increase in viral protein expression. Expression of FLAG-tagged transfected PKG1β (top panel) and HIV-1 viral proteins are shown by Western blotting.

**Figure 2:**
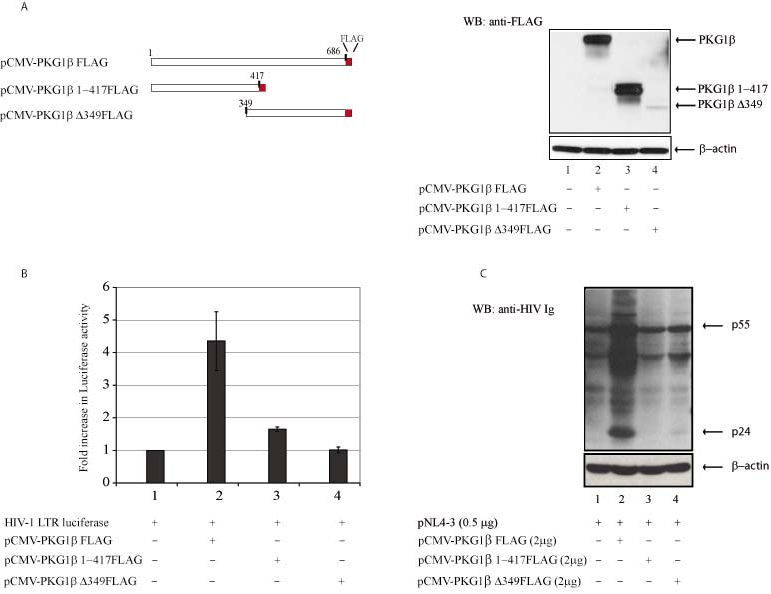
Intact PKG1 β is required for activation of gene expression. A) Schematic representations are shown of full length PKG1β (pCMV-PKG1β FLAG) and two deletion mutants, pCMV-PKG1β 1–417 FLAG and pCMV-PKG1β Δ349FLAG. Transfected cell lysates (right panel) were analyzed by Western blotting using anti-FLAG antibody for expression. B) Full length PKG1β, but not its deletion mutants, activated LTR-luciferase expression. HeLa cells were transfected with the indicated plasmids. C) Full PKG1β, but not its deletion mutants, activated pNL4-3-expression. Plasmids were transfected into HeLa cells as indicated. C- and N-terminus deletions of PKG1β resulted in loss of activity.

To address how cGMP/PKG might influence HIV-1 LTR-directed expression, we transfected HeLa cells with a LTR-luciferase reporter with or without a Tat-plasmid [6-9]- and assayed for expression with or without simultaneously co-transfecting a PKG1β-expression vector [10]- (Figure 1). We found that PKG1β-alone activated reporter expression by approximately 4 fold (also see Figure 2B) while Tat-alone activated expression by >45 fold (Figure 1A). Co-transfection of LTR-luciferase + Tat + PKG1β activated expression cumulatively to >175 fold (Figure 1A). These results are consistent with PKG1β inducing LTR-driven expression whether in the absence or presence of Tat.

To verify more physiologically the above LTR-reporter assay, we next checked the effect of PKG1β on an HIV-1 infectious molecular clone, pNL 4-3 (Figure 1B). Here, increasing amounts of PKG1β-plasmid were transfected into cells with a constant level of pNL 4-3. Viral proteins expressed from the HIV-1 molecular clone were then measured. Using HIV-specific hyper-immune sera in Western blots, we observed that PKG1β increased pNL 4-3 expression in a dose dependent manner (Figure 1B, lanes 2 – 6). To investigate whether the intact function of PKG1β was needed for this activity, we created two loss-of-function PKG1β deletion mutants (Figure 2A). Both deletion mutants were unable to activate either a LTR-reporter (Figure 2B) or an HIV-1 infectious molecular clone (Figure 2C).

Optimal PKG activity is dependent on activation by cGMP [11]. While over expression of exogenously transfected PKG offered significantly measureable effects (Figures 1, 2), we wished to understand next how cell endogenous PKG might act mechanistically in response to cGMP treatment. Elsewhere, it was reported that NF-κB p65, p52, and p50 are substrate proteins activated by PKG-mediated phosphorylation. Because expression of the HIV-1 LTR is regulated by NF-κB [12,13], we asked if cGMP activated NF-κB in our experimental.

**Figure 3:**
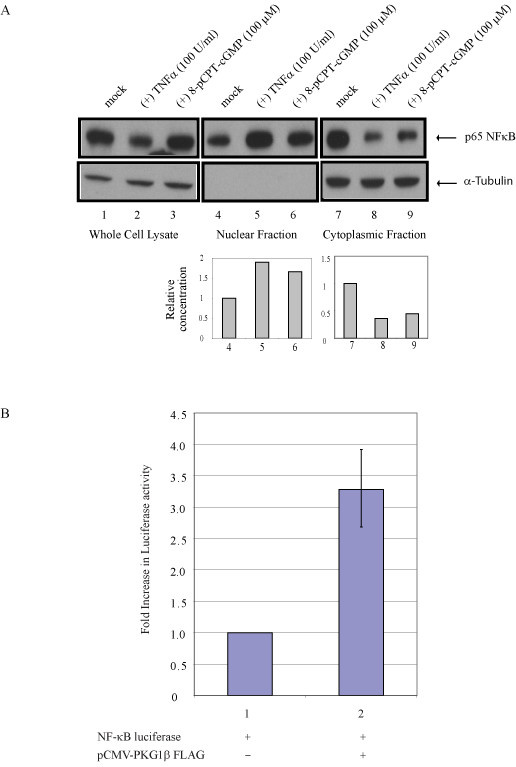
Treatment of HeLa cells with cGMP agonist, 8-(4-chlorophenylthio)guanosine 3'.5'-cyclic monophosphate (8-pCPT-cGMP), increased NF-κβ p65 in the nucleus. A) HeLa cells were mock treated or treated with TNFa or 8-pCPT-cGMP as indicated. Nuclear and cytoplasmic fractions were collected and assayed for NF-κβ p65 by Western blotting. Both TNFα and 8-pCPT-cGMP were found to increase nuclear NF-κβ p65 while commensurately decreasing cytoplasmic NF-κβ p65. Western blotting for tubulin controlled for subcellular fractionations. Quantifications of relative distributions of NF-κβ p65 are shown at bottom. B) PKG1β increases expression of NF-κB dependent luciferase. HeLa cells were transfected with NF-κB luciferase reporter plasmid in the presence or absence of co-transfected PKG1β expression plasmid. Cells were harvested 24 hours post-transfection, and cell lysates were assayed for luciferase activity as indicated.

To assess if NF-κB was activated by cGMP, we assayed for enhanced nuclear localization of NF-κB protein p65, which is one measure of activation [14,15]. We treated cells with a cGMP agonist, 8-pCPT-cGMP, and compared results to cells treated with a known NF-κB activator, tumor necrosis factor alpha, TNFα. When cytosolic and nuclear p65 proteins were assayed, we observed that 8-pCPT-cGMP behaved quantitatively very similarly to TNFα in inducing increased NF-κB p65-translocation into the nucleus (Figure 3A). This result supports that PKG-activation of HIV-1 (Figures 1, 2) acts through an NF-κB-mediated process. To confirm that PKG1β can directly activate NF-κβ activity, we transfected a NF-κβ luciferase reporter into cells with or without a co-transfected PKG1β expression plasmid (Figure 3B). Results from this assay showed that NF-κβ-dependent luciferase activity was indeed elevated by PKG1β.

**Figure 4:**
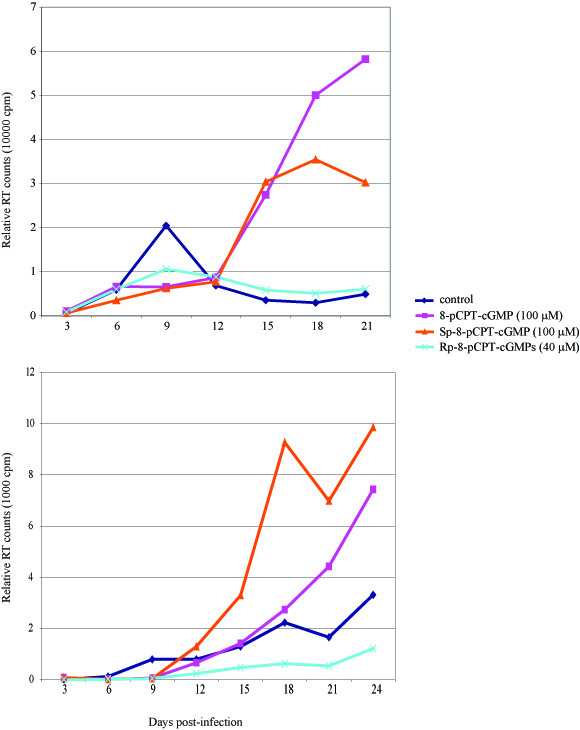
Effect of cGMP analogues on pAD8 replication in monocyte derived macrophages (MDM's). MDM's were cultured in RPMI medium containing 10% human serum, infected 5-7 days post culture with HIV-1 AD8 virus, and cells were treated at time of infection with cGMP agonists -8-pCPT-cGMP and Sp-8-pCPT-cGMP or cGMP antagonist (Rp-8-pCPT-cGMPs). Culture supernatants were collected every third day and assayed for virus production by RT assays. cGMP agonists increased virus production from AD8 infected MDM's while cGMP antagonists suppressed virus production.

A more direct verification of cGMP/PKG's role in HIV-1 replication can be established using chemical agonists and antagonists of this pathway seeking for effects on viral infection. Because the NO/cGMP signaling pathway has been reported to function potently in cells of macrophage lineage, we performed infection of monocyte derived macrophages (MDMs) using HIV-1 strain AD8 [16,17]. MDMs infected with AD8 were treated individually with three different drugs. We employed two cGMP agonists (8-pCPT-cGMP, and Sp-8-pCPT-cGMP) and one cGMP antagonist (Rp-8-pCPT-cGMPs) and monitored their impact on HIV-1 replication. Informatively, in two separate experiments, both agonist drugs increased virus replication over control-treated infection, while the cGMP-antagonist drug decreased (or did not affect) virus replication (Figure 4).

Here, we report evidence that both in the absence and presence of Tat the cGMP/PKG pathway can serve to modulate HIV-1 expression/replication. Understanding how HIV-1 LTR expression is affected by ambient cellular pathways [18-20] may help to address potential approaches for treating latent HIV-1 infection [21]. The current findings may be important because cGMP is a ubiquitous second messenger that affects multiple cellular pathways in most, if not all, cells. Accordingly, cGMP-influenced pathways are likely to interdigitate with some of the signaling routes utilized by HIV-1 in infected cells [22]. Additionally, because many cGMP chemical agonists and antagonists are available [23,24], practical chemotherapeutic interventions in these pathways (if they should be useful for anti-viral purposes) could be amenable.

## Competing interests

The author(s) declare that they have no competing interests.

## Authors' contributions

JHL and VY carried out the experiments. JHL, VY, and KTJ conceived of the study and wrote the manuscript.
